# Investigation of an outbreak of novel hepatitis of unknown aetiology in children and adolescents, Ireland, 2021 to 2023

**DOI:** 10.2807/1560-7917.ES.2025.30.14.2400536

**Published:** 2025-04-10

**Authors:** Emer Liddy, Niamh Murphy, Jolita Mereckiene, Emer Fitzpatrick, Annemarie Broderick, Róisin Egan, Tiarnán Fallon Verbruggen, Julie-Anne Houlihan, Christine Campbell, Michael Carr, Gabriel Gonzalez, Jonathan Dean, Richard Hagan, Cillian De Gascun, Suzanne Cotter, on behalf of the Incident Management Team

**Affiliations:** 1 Health Protection Surveillance Centre (HPSC), Dublin, Ireland; 2 Department of Paediatric GI, Liver and Nutrition, Children’s Health Ireland, Crumlin, Dublin, Ireland; 3 National Virus Reference Laboratory, University College Dublin, Dublin, Ireland; 4 International Collaboration Unit, International Institute for Zoonosis Control, Hokkaido University, Sapporo, Japan; 5 Japan Initiative for World-leading Vaccine Research and Development Centers, Hokkaido University, Institute for Vaccine Research and Development, Sapporo, Japan; 6 Molecular Biology and Genetics Department, Irish Blood Transfusion Service, Dublin, Ireland; 7 The members of the Incident Management Team are acknowledged at the end of the article

**Keywords:** paediatric hepatitis, AAV2, HAdV, adeno-associated virus 2, adenovirus, HLA, autoantibodies

## Abstract

An outbreak of severe acute hepatitis of unknown aetiology in children (HUAC) was reported by the United Kingdom (UK) in spring 2022. Within days, a corresponding increase was identified in Ireland. A multi-agency incident management team (IMT), led by the Health Protection Surveillance Centre (HPSC), established a national case definition, trawling questionnaire, testing protocol and communications plan. Between 1 October 2021 and 12 May 2023, 44 probable and three possible cases of HUAC were identified in Ireland with a median age of 3 years. Adeno-associated virus 2 (AAV2), detected in 18 of 31 probable cases, and SARS-CoV-2 antibodies in 22 of 37 of probable cases were the most common infectious agents, followed by human herpes virus 7 (18/33) and adenovirus (20/44). Immunological findings included the human leukocyte antigen (HLA) class II HLA-DRB1*04:01 allele in 17 of 32 cases. Autoantibodies were found in 15 of 40 patients. Our findings corroborate those of the UK, which suggested a link between HUAC and AAV2 and another virus, in children predisposed due to presence of a particular HLA class II type. Close collaboration with the UK, the European Centre for Disease Prevention and Control (ECDC) and World Health Organization (WHO) was invaluable in the investigation.

**Key public health message**

**What did you want to address in this study and why?**

An unusual outbreak of liver inflammation of unknown cause in children and adolescents was identified in several countries in Europe, including Ireland, in spring 2022. This outbreak resulted in severe illness in a very young and vulnerable population group. We analysed the data from affected patients to understand the cause of this outbreak.

**What have we learnt from this study?**

The cause of this outbreak was complex and probably involved infection with more than one pathogen in addition to underlying genetic susceptibility i.e. presence of class II HLA-DRB1*04:01 allele. Control measures to curb COVID-19 resulted in changes to typical transmission patterns of common infectious diseases among children and adolescents. This change in transmission patterns may have contributed to this outbreak.

**What are the implications of your findings for public health?**

Close collaboration between public health authorities at a national level, facilitated by the European Centre for Disease Prevention and Control (ECDC) and World Health Organization (WHO), was crucial for the investigation and management of this event in several countries, particularly for smaller countries such as Ireland. Further research is needed to understand the cause of this outbreak to learn for future incidents that may arise.

## Background

Hepatitis of unknown aetiology in children (HUAC) contributes to a substantial proportion of acute liver failure (ALF) cases in children [1-3]. A British Paediatric Surveillance Unit study, based on data collected from both the United Kingdom (UK) and Ireland from 2014 to 2015, categorised 23% of cases of acute hepatitis as not having hepatitis A–E virus (non-A–E hepatitis) and 33% as having no identifiable aetiology [3]. There is no clear definition for HUAC, but the term HUAC is used to describe a syndrome involving severe clinical and biochemical liver injury which may or may not lead to ALF, defined by an international normalised ratio (INR) > 2 with or without encephalopathy or an INR > 1.5 in the presence of encephalopathy (INR not correctable by vitamin K).

An outbreak of severe acute HUAC emerged in Europe in spring 2022, at a time when COVID-19 lockdowns were relaxed or discontinued in many countries [4,5]. In late March 2022, Scottish public health authorities were alerted to five children aged 3–5 years who had presented to hospital in Glasgow with severe acute hepatitis in a 3-week period, exceeding the expected yearly number of such cases for the entire country. The World Health Organization (WHO) was informed, and an alert issued to all countries. By 12 April, 13 cases had been identified in Scotland in addition to ca 60 cases in the rest of the UK [6,7].

For the purposes of reporting during the outbreak we describe, the European Centre for Disease Prevention and Control (ECDC) selected a value exceeding 500 IU/L as a cutoff for aspartate transaminase (AST)/alanine transaminase (ALT), indicating the relative severity of liver inflammation (> 10 times the reference value) [5]. Though mild elevation in AST/ALT is often seen as part of a viral illness or mild drug-induced disease, it is thought to be rare to develop a more profound liver inflammation, particularly in those who are negative for hepatitis A–E viruses. Acute severe hepatitis may be the presenting feature of more severe drug-induced liver injury or of more chronic liver disease such as autoimmune liver disease or Wilson disease. Other inborn errors of metabolism may also present in this manner.

Data from January to April 2022, published by one of the three paediatric UK liver transplant units, described 13 cases of HUAC, in comparison with 1–5 cases admitted during the same time period in the preceding 10 years [8]. Although no incidence data for HUAC were published before 2022 in Ireland or most other jurisdictions, clinicians from the national paediatric hepatology unit reported such cases as rare, with, on average, perhaps one patient with ALF from HUAC requiring liver transplant every second year in Ireland (data not shown).

## Outbreak detection

On receipt of the initial alert on 6 April 2022, the Health Protection Surveillance Centre (HPSC) in Ireland cascaded the information to Irish clinicians requesting immediate notification of any similar cases in Ireland. On 7 April, clinicians at the National Paediatric Liver Centre (NPLC) Children’s Health Ireland (CHI), Crumlin, reported a similar increase, with three cases detected in a recent 4-week period. This also exceeded the expected annual caseload of acute non-A–E hepatitis in children in Ireland [3].

We describe here the epidemiology of cases of HUAC identified in Ireland, between 1 October 2021 (date recommended by WHO based on the first identified cluster of cases in the United States (US)) [9] and 12 May 2023 (date the Irish national Incident Management Team (IMT) closed the active investigation), and the work undertaken by the national IMT.

## Methods

### National Incident Management Team (IMT)

Led by HPSC, a multi-agency IMT was established to investigate this outbreak in Ireland. The first meeting took place on 12 April 2022. Terms of reference and surveillance needs were agreed. The IMT included representatives from the National Virus Reference Laboratory (NVRL), microbiology, NPLC at CHI, public health, infectious diseases, pathology, general practice, the Office of the Chief Clinical Officer, toxicology and the Health Service Executive (HSE) Communications. The IMT structure fostered a collaborative investigation. Close engagement with the UK and international collaboration were prioritised.

Disease clusters and changing patterns of illness of public health concern are notifiable under the Irish infectious disease legislation enabling immediate response to request for notification of HUAC upon receipt of the international alert [10]. To ensure standardisation of surveillance, the IMT agreed on a national case definition, trawling questionnaire and testing protocol similar to that proposed by the UK and WHO and a communications plan. Regular epidemiological reports were produced for the IMT and HPSC website [11] as well as bespoke reports on human adenovirus (HAdV) detection rates from laboratory reporting, review of hospital discharge coding for HUAC cases using ECDC recommended codes, cross-check of hospital discharge coding used for Irish cases and an IMT after-action review.

### Case definition

The national IMT agreed on the following case definition: hepatitis of unknown aetiology (hepatitis A–E viruses excluded) in children and adolescents aged < 17 years at time of onset 1 October 2021–12 May 2023. A case was defined as probable if serum transaminases were > 500 IU/L (AST or ALT) and as possible if AST or ALT were 200–500 IU/L and other results indicated a cholestatic picture (conjugated bilirubin > 5 µmol/L). Cases of any age presenting with an acute hepatitis (non-A–E hepatitis virus) and who were a close contact of a probable case since 1 October 2021, were defined as epidemiologically linked. These case definitions matched those used by WHO and ECDC except for the possible case category, which was unique to Ireland. Cases with alternative explanations for their clinical presentation were discarded from the investigation. All cases underwent a full standard workup for severe acute hepatitis according to age. This included history of drug and toxin exposure, investigation for viral causes, screening for autoimmune liver disease, Wilson disease (in those aged > 3 years) and inborn errors of metabolism. Evidence for haemophagocytic lymphohistiocytosis and other haematological disorders and malignancies was also sought. A trawling questionnaire was completed for each case by regional public health authorities or the paediatric team.

### Identification of outbreak cases

Active case finding was done through regular communications to clinicians nationwide following the initial alert on 6 April, requesting that any cases meeting the case definition be reported to the regional Medical Officer of Health (Public Health authority at regional level), who would then notify HPSC, and also to discuss with the NPLC. Data on clinical presentation, laboratory results, exposures and outcome were collected by HPSC using a standardised questionnaire. Later in the investigation, clinicians at the NPLC conducted a retrospective case finding review to identify any cases who presented to their hospital from 1 October 2021, before the identification of the outbreak in Europe.

### Laboratory analyses

The WHO interim guidance on testing was followed, where possible, and samples collected from all patients included blood, faecal and respiratory samples [12]. Liver function tests (including AST, ALT, bilirubin) were done in the hospital of admission. Viral testing was done in the NVRL, apart from human herpesvirus (HHV)-7 which was referred to the United Kingdom Health Security Agency (UKHSA) Virus Reference Laboratory, London. Molecular testing with PCR was done for adeno-associated virus (AAV)2, HAdV, HAdV species F40/41 (in NVRL), cytomegalovirus (CMV), Epstein-Barr virus (EBV), enterovirus, hepatitis A virus (HAV), hepatitis C virus (HCV), hepatitis E virus (HEV), HHV-6, HHV-7, herpes simplex virus (HSV) type 1, parechovirus and parvovirus B19. Serological testing was done for CMV, EBV viral capsid antigen (VCA), nuclear antigen 1 of EBV (EBNA), HAV, hepatitis B virus (HBV), HCV, HEV, *Leptospira* spp., parvovirus B19 and severe acute respiratory syndrome coronavirus 2 (SARS-CoV-2).

Plasma samples were tested by real-time quantitative (q) PCR for AAV2 viral protein (VP) 1 and non-structural protein (NSP) targets using the TaqMan Fast Advanced Master Mix (Thermo Fisher, Waltham, US) on the ABI7500 FAST platform (Applied Biosystems, Thermo Fisher), as described previously [13]. In addition, plasma samples for probable cases were also genotyped using high-resolution typing for all major HLA loci (*HLA-A*, *HLA-B*, *HLA-C*, *HLA-DRB1*, *HLA-DRB3*, *HLA-DRB4*, *HLA-DRB5*, *HLA-DQA1*, *HLA-DQB1*, *HLA-DPA1* and *HLA-DPB1*). Testing for HLA was done in the Irish Blood Transfusion Services, National Blood Centre, Dublin.

Routine autoantibody testing was performed, as was quantification of immunoglobulin (IgG) levels in CHI [14]. Autoantibodies quantified included antinuclear antibody (ANA), smooth muscle antibody (SMA), and liver-kidney microsomal (LKM) antibody.

### Capacity to detect human adenovirus in Irish laboratories

As HAdV infection is not notifiable in Ireland and there were no data on prevalence, HPSC undertook an ad hoc assessment of the capacity of the Irish hospital laboratories to test for HAdV and requested data on detections since 2017 to understand detection trends over time. In early May 2022, a link to an electronic questionnaire using Qualtrics (https://www.qualtrics.com/) was sent to 27 publicly funded laboratories. The questionnaire contained questions on testing capacities for HAdV, sample types (respiratory, faecal/blood/serum samples), analytical methods, age criteria for testing and ability to perform serotyping. The survey was completed by laboratories between 24 May and 30 June 2022). Laboratories with testing capacity were asked to provide their data using an Excel template for the period January 2017–May 2022. Age-specific detection rates were calculated using census data.

### Hospital discharge coding

In June 2022, ECDC published a protocol for assessment of exceedances of HUAC in the European Union/European Economic Area (EU/EEA) countries [15]. These countries were requested to extract data using 11 pre-defined International Classification of Diseases (ICD)-10 codes (https://icd.who.int/browse10/2019/en) and submit these data to ECDC for analysis. In Ireland, the IMT noted that the number of discharges extracted was smaller than the number of HUAC cases notified to the IMT for this time period. A review was therefore undertaken to assess the ICD-10 coding on discharge for the HUAC cases identified in Ireland.

In November 2022, HPSC requested information from Irish hospitals where probable and possible HUAC cases were admitted, regarding the ICD-10 codes assigned to these cases on discharge as either a primary or secondary diagnosis. The ICD-10 codes received from hospitals were cross-referenced with the IEBook (10th Edition ICD-10-AM/ACHI/ACS classification, available at https://hpo.ie/iEbook [16].

### Data analysis

Case-based data were inputted into an MS Access database and analysed by HPSC staff. Data were analysed by sex, age group, ethnicity, COVID-19 vaccination status, laboratory results and clinical outcome. We present detailed data relating to the investigations of cases meeting the probable case definition. Data analysis was done using Microsoft Excel for Microsoft 365 and Stata version 15.1 (https://www.stata.com/). Pearson’s chi-square test, with corresponding probability value (p value) and 95% confidence intervals (CI), was used to test for statistically significant associations between specific HLA alleles and autoimmune phenotype, and between illness severity and autoimmune phenotype. Wilcoxon rank-sum test and t-test were used to test for differences in liver function test results between patients with and without autoimmune phenotype.

## Results

### Descriptive epidemiology

Forty-four probable and three possible cases of HUAC were identified in Ireland. Two of the probable cases were epidemiologically linked, both attending the same child-care facility.

**Figure 1:**
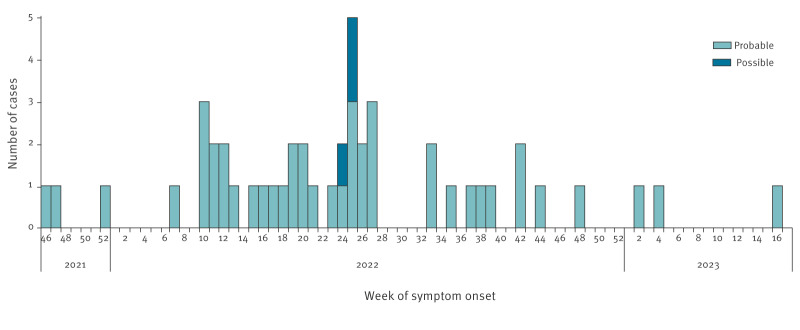
Distribution of notified cases of hepatitis of unknown aetiology in children and adolescents aged < 17 years, by week of onset of symptoms, Ireland, 1 October 2021–12 May 2023 (n = 47)

Most children (27 probable and all three possible cases) presented with symptoms during a 17-week period 6 March–9 July 2022 (weeks 10–27). There was a steady decline in cases from that time onwards, with only four probable cases presenting 1 December 2022–22 April 2023 (Figure 1).

The following results reported relate to the 44 probable cases only. The median age was 3 years (range: 0–15 years) with equal sex balance (Table). Forty-three cases were hospitalised and eight required treatments in intensive care unit (ICU). Two cases underwent liver transplant, and three cases were transferred to the UK for transplant assessment but did not require transplant. One patient died.

**Table:** Characteristics of probable cases of hepatitis of unknown aetiology in children and adolescents aged < 17 years, Ireland, 1 October 2021–12 May 2023 (n = 44)

Characteristics	Cases (n)	%
Age (years)		
< 1	6	13.6
1–4	25	56.8
5–11	11	25.0
12–16	2	4.5
Median age	3	NA
Sex		
Male	22	50.0
Female	22	50.0
Ethnicity		
White Irish	37	84.1
Other	3	6.8
Unknown	4	9.1
SARS-CoV-2 vaccination		
Vaccinated	3	6.8
Not vaccinated	33	75.0
Unknown	8	18.2
Hospitalisation (n = 43)		
Other than intensive care unit	35	79.5
Intensive care unit	8	18.2
Transplant		
Liver transplant	2	4.5

NA: not applicable; SARS-CoV-2: severe acute respiratory syndrome coronavirus 2.

Cases were identified in most regions of the country with no evidence of any rural-urban divide. Several cases reported gastrointestinal (18/43) or respiratory symptoms (15/43) in the weeks or months before presentation. The most reported symptoms at the first presentation were malaise (29/35), pale stools (25/33), abdominal pain (29/44), vomiting (28/44), jaundice (26/44), fever (20/44), nausea (13/31) and diarrhoea (17/44).

### Laboratory results

**Figure 2:**
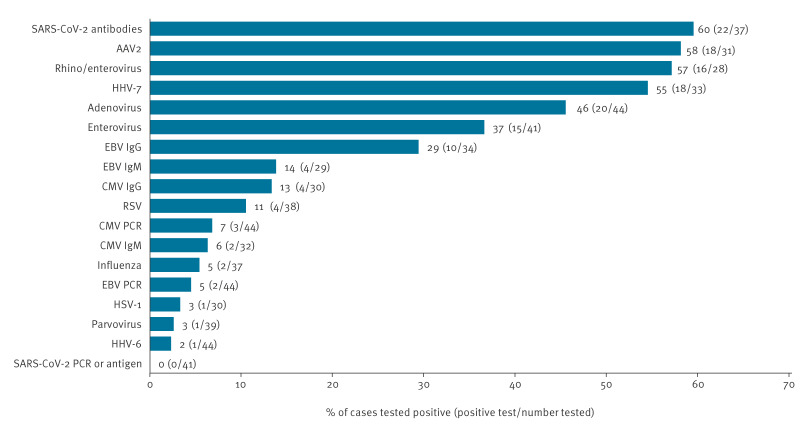
Percentage of probable cases of hepatitis of unknown aetiology in children and adolescents aged < 17 years positive for each organism where results reported, Ireland, 1 October 2021–12 May 2023 (n = 44)

AAV: adeno-associated virus; CMV: cytomegalovirus; EBV: Epstein-Barr virus; HHV: human herpesvirus; HSV: herpes simplex virus; RSV: respiratory syncytial virus; SARS-CoV-2: severe acute respiratory syndrome coronavirus 2.

Testing for pathogens on the recommended testing panel was not possible for all probable cases due to insufficient residual samples. All 44 probable cases were tested for HAdV. The results for the other pathogens were as follows: SARS-CoV-2 antibodies (22/37), AAV2 (18/31), rhinovirus/enterovirus (16/28), HHV-7 (18/33), adenovirus (20/44) and enterovirus (15/41) (Figure 2). Autoantibody testing was done for 40 of 44 probable cases. Of the 40 cases tested, 15 cases were positive for either LKM or ANA/SMA antibodies; seven cases for LKM antibodies and 11 cases for ANA/SMA antibodies, while IgG was elevated in 5 of 39 cases. Though suspicious for autoimmune hepatitis (AIH) (especially in case of LKM positivity), the clinical characteristics of cases with detectable autoantibodies were indistinguishable from the rest of the cohort, median ALT/AST of 1,560/1,660. Two of 15 cases met definition for ALF. More details can be seen in Supplementary Table S1.

Typing of HLA was done for 32 cases, with class HLA-DRB1*04:01 allele detected in 17 cases. The class II HLA allele subtypes more commonly associated with AIH, HLA-DRB1*03:01 was found in nine and HLA-DRB1*07:01 in seven cases. The HLA types for the two epidemiologically linked cases were different. More details are presented in Supplementary Table S1.

### Human adenovirus detection rates

Of the 27 clinical laboratories invited to participate in the survey, 21 responded, 10 completed the data template and returned data to HPSC. Twenty laboratories were able to test for HAdV, while one laboratory referred all samples to the NVRL for testing. Of the 20 laboratories testing for HAdV, 11 routinely tested faecal samples, while the remaining nine did not test routinely.

**Figure 3:**
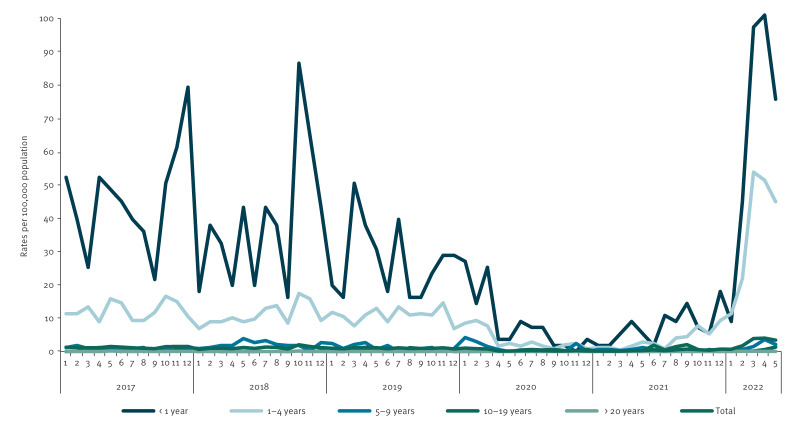
Monthly detection rates of adenovirus from faecal samples in 10 laboratories, by age, Ireland, 2017–2022 (n = 109,522)^a^

^a^ Adenovirus was detected from 3,143 samples.

Source: Publicly funded laboratories in Ireland, July 2022.

Between March 2020 and November 2021, HAdV faecal detection rates were low compared with previous years (2017–2019), notably in children aged < 1 and 1–4 years. By December 2021, detection rates had started to increase in both age groups, peaking at 100.9 per 100,000 and 51.4 per 100,000, respectively, by April 2022. The increase in HAdV detection rates, particularly in faecal samples, occurred at the same time as the peak in cases of HUAC in Ireland (Figure 3).

### Hospital discharge coding

Discharge coding data were available for 30 probable cases, including 41 separate discharge events as some cases had more than one discharge event due to hospital transfer or readmission. Analysis of the data demonstrated that 22 of these cases were assigned at least one of the ECDC selected ICD-10 codes. The most common of these codes were B17.9 (Acute viral hepatitis, unspecified) assigned to 14 of 41, followed by B17.8 (Other specified acute viral hepatitis) assigned to 7 of 41.

## Outbreak response measures

Based on the initial hypothesis from the UK of possible HAdV association, communications issued focussed on generic hygiene measures (hand washing, respiratory etiquette, staying at home if ill with respiratory or gastrointestinal illness) and seeking clinical assessment if jaundice was identified. Information for parents was published online by HPSC and HSE, which highlighted standard personal (handwashing before and after toileting and before eating, covering coughs and sneezes and washing hands) and environmental hygiene measures such as regular cleaning and decontamination of surfaces and items frequently handled by individuals) to prevent transmission of respiratory or gastrointestinal infections in settings frequented by young children.

## Discussion

A marked exceedance of cases of HUAC was identified in Ireland, with a peak in spring–summer 2022. Ireland had the fourth highest number of cases in Europe, and third highest in the EU/EEA countries, despite being one of the least populous countries in these regions. The two countries in the EU with higher case numbers than Ireland, Spain and Italy, reported roughly twice the number of cases, yet have populations ca 9 and 10 times the size of Ireland [17]. Ireland’s caseload in comparison with the UK was similar, when population size is accounted for [8].

We present complete data for this syndrome from October 2021 to May 2023, including results of HAdV, AAV2, SARS-CoV-2, autoimmune hepatitis-associated antibodies and HLA-DRB1 status of cases. These data are strengthened by the reporting here of HAdV national trends as well as ICD-10 coding for cases. A temporal association is shown here between an increase in HAdV rates nationally in young children (aged ≤ 4 years) and cases of HUAC. This finding is also supported by a previous study which reported a high correlation between HAdV-F41 and AAV2 circulation in the community from January to July 2022 peaking in April 2022, demonstrated through testing of wastewater in Ireland [13].

The Irish findings also corroborate those published in the UK, which suggest a link between this syndrome and AAV2 along with another helper virus, such as HAdV or a herpes virus, in children who were predisposed to severe disease due to presence of a particular HLA class II type [18,19]. Of the cases in our study tested for *HLA-DRB1*, 17/32 (53%) were positive for the DRB1*04:01 allele, which is more than the 20% reported in an Irish bone marrow registry study [20]. Identification of this genetic predisposition suggests that there may be an underlying autoimmune predisposition in children who tested positive for the HLA-DRB1*04:01 allele. This suspected autoimmune susceptibility with either HLA subtypes or autoantibody positivity led to the use of steroid treatment in nine of the Irish cases following liver biopsy which was suggestive of an autoimmune injury. Of note, there was no increase in proportion of HLA-DRB1*03:01 (nine cases) and *07:01* (seven cases) in this cohort – alleles found more commonly in association with type 1 and 2 AIH – compared with background population prevalence (33% and 30%) [20]. The use of steroids in children with indeterminate ALF is standard practice in a small number of centres but not usually based on evidence of autoimmunity. The autoimmune phenotype identified in this cohort provided some rationale for the cautious use of steroids in a selected number of children. No child who received steroids was transplanted or died.

The proportion of SARS-CoV-2 antibody test positivity (22/37) was similar to that of the age-matched background population (73.3% of 0–4-year-olds tested in June 2022) [21], and no cases tested positive for SARS-CoV-2 RNA at time of presentation. This supports the hypothesis that SARS-CoV-2 was not linked directly to the aetiology of this syndrome.

This syndrome of HUAC may be related to a post-lockdown phenomenon and increased circulation of viruses when social mixing returns to normal with an increase in some infections. Further research is needed to understand this phenomenon and its full impact in order to learn for future pandemic response.

Although the aetiology of this syndrome is not yet fully deciphered, results of investigations completed thus far have revealed it as complex and most probably multifactorial combining infection and host genetics. The leading hypothesis to date is that AAV2, likely acquired as a co-infection with HAdV and/or HHV-6B triggered an immune-mediated liver disease in children who were genetically predisposed because of their HLA class II status [18,19].

Most cases appear to have occurred sporadically, with very few examples of epidemiologically linked cases [6,22,23]. However, considering the likely multifactorial aetiology, deciphering transmission dynamics is inherently complicated.

As HUAC had not been notifiable or included within routine surveillance systems before 2022, and as it remained a relatively rare occurrence with low case numbers, it was particularly challenging for the investigating public health authorities to identify whether and where there was a true exceedance of this syndrome.

A study in April 2022 showed no overall increase in HUAC in Europe; however, of 34 liver centres included in the study, 22 reported no increase, while 12 reported a suspicion of increase but had not documented a rise in cases [24]. Another study from April 2022 identified an increase in HUAC in 2022 compared with the previous 5 years in five of 17 countries in Europe surveyed [25]. Italy was one of the five countries with an identified increase in HUAC cases in this survey: however, a subsequent Italian study including further data concluded that the 2022 Italian data were similar to those observed in previous years [26]. Germany was one of the 12 European countries with no increase in HUAC cases in this study, yet a 30-year retrospective study from northwestern Germany published in December 2022 concluded that there was evidence of an increase in incidence of HUAC from 2019 onwards [27]. Similarly, despite initial reports from Alabama in the US [9] which identified an increase in cases of HUAC in late 2021, subsequent evaluation of data at a national level has shown no evidence of an increase in HUAC in the US compared with pre-pandemic levels [28]. These examples highlight the difficulty in identifying exceedances of this syndrome and the need for more published data on its incidence over time. Ireland notably is not included in either of the 2022 pan-European studies.

A national multi-disciplinary IMT which brought together national experts from public health, paediatrics, pathology, microbiology/virology, infectious diseases, toxicology, and communications is the ideal framework through which a novel syndrome of unknown aetiology is investigated. Close collaboration with international partners and neighbouring countries is crucial.

Active syndromic surveillance can result in overreporting of cases meeting the case definition. It was not possible to test all cases for the full panel of virus testing as residual plasma/serum samples from the early cases were not available for testing at a later stage in the investigation. The absence of a unique patient identifier in Ireland means that hospital discharge data do not correlate to cases. The pathogens associated with this outbreak are not notifiable. The increase in detection rates of HAdV in faecal samples in the first 5 months of 2022 may reflect a bias in detection rates. However, as HAdV detections in wastewater also increased during this period, we believe our results are an accurate reflection of increased circulation [13]. As a result of this incident and the ensuing investigation, clinicians (particularly paediatricians) are now primed to notify unusual clusters of disease to public health. The value of the multi-disciplinary investigation enabled rapid mobilisation of expertise to this investigation.

## Conclusions

The occurrence of this outbreak of HUAC in young children in Ireland in spring-summer 2022 was unprecedented. Early recognition and reporting resulted in an immediate national response and active case finding. Our investigation supports the findings reported by other countries of a possible association with AAV2, and possible co-infection with HAdV triggering an immune mediated liver disease in children with a genetic susceptibility. This report demonstrates that outbreaks of unknown aetiology can be investigated, despite their complexity, if adequate resources and expertise is mobilised – aided by information sharing across jurisdictions and disciplines.

## Supplementary Data

521000 Supplementary Material
