# Supplementary material for: Investigation of an outbreak of novel hepatitis of unknown aetiology in children and adolescents, Ireland, 2021 to 2023
Source: Euro Surveill. 2025 Apr 10;30(14):2400536. doi: 10.2807/1560-7917.ES.2025.30.14.2400536 (PMC11987496; doi:10.2807/1560-7917.ES.2025.30.14.2400536)
Supplement: Supplementary Material [file 24-00536_COTTER_Supplement.pdf]

This supplementary material is hosted by Eurosurveillance as supporting information alongside the article ['Irish investigation of novel Hepatitis of unknown aetiology outbreak in children, 2021-2023'], on behalf of the authors, who remain responsible for the accuracy and appropriateness of the content. The same standards for ethics, copyright, attributions and permissions as for the article apply. Supplements are not edited by Eurosurveillance and the journal is not responsible for the maintenance of any links or email addresses provided therein.

Supplementary Table

Table S1. Characteristics of probable cases categorised as AIH type I or type II compared to cases who did not test positive for LKM, ANA or SMA[a], 1st October 2021- 12th May 2023, Ireland. (n=40)

Characteristics	All probable cases		Autoimmune hepatitis I or II		Autoantibody tests done, case not positive for LKM, ANA or SMA	
Number of probable cases	44		15/40	38%	25/40	63%
Median age (years)	3		3		3	
Females	22		8/15	53%	12/25	48%
Median ALT	1697		1660		1811	
Median AST	1875		1560		2022	
Median Bilirubin	79		80		61	
Acute liver failure	9/44	20%	2/15	13%	6/25	24%
Cholestatic	25/40	63%	9/15	60%	14/25	56%
HLA-DRB1*04:01:01	17/32	53%	6/11	55%	11/20	55%
HLA-DRB1*07:01:01	7/32	22%	2/11	18%	5/20	25%
HLA-DRB1*03:01:01	9/32	28%	5/11	45%	4/20	20%

a = LKM - Liver kidney microsome, ANA – antinuclear antibodies, SMA – smooth muscle antibodies.
